# Ecofriendly spectrophotometric methods for simultaneous determination of remdesivir and moxifloxacin hydrochloride as co administered drugs in corona virus treatment

**DOI:** 10.1038/s41598-024-83049-4

**Published:** 2025-01-11

**Authors:** Eman A. Madbouly, Abdalla A. El-Shanawani, Sobhy M. El-adl, Ahmed S. Abdelkhalek

**Affiliations:** Department of Medicinal Chemistry, Faculty of Pharmacy, Zagazig University, Zagazig, Egypt

**Keywords:** Covid-19, Remdesivir, Moxifloxacin hydrochloride, Spectrophotometry, Medical research, Chemistry

## Abstract

Remdesivir and moxifloxacin hydrochloride are among the most frequently co-administered drugs used for COVID-19 treatment. The current work aims to evaluate green spectrophotometric methodologies for estimating remdesivir and moxifloxacin hydrochloride in different matrices for the first time. The proposed approaches were absorbance subtraction, extended ratio subtraction and amplitude modulation methods. In order to determine the absorbance of the investigated medications in combination at the isoabsorptive point, the pure moxifloxacin hydrochloride absorbance factor is applied using the absorbance subtraction method, which modifies the zero absorption spectra of the drugs under investigation at the isoabsorptive point (229 nm). The spectrum of moxifloxacin hydrochloride is more extended in the plateau area between 340 and 400 nm, where remdesivir exhibits no absorption. So, also, the ratio spectra were successfully manipulated for quantification of the two drugs. Regarding the pharmacokinetic profile of remdesivir (Cmax 4420 ng/mL) and moxifloxacin hydrochloride (Cmax 3.56 µg/mL), the proposed methods were effectively used to spectrophotometrically determine remdesivir and moxifloxacin hydrochloride in plasma matrix. The new approach was validated using the ICH guidelines for specificity, linearity, precision, and accuracy. The greenness of the reported methodologies was evaluated using two metrics: the analytical eco-scale and the green analytical procedure index.

## Introduction

On March 11, 2020, WHO proclaimed COVID-19 a global pandemic. According to the WHO estimate in May 2024, up to 7 million people have died as a result of COVID-19^1^. The swift transmission and devastating consequences of the virus have prompted scientists worldwide to explore antiviral medications to slow down the virus’s transmission and facilitate the recovery of patients. The disease primarily affects the lower respiratory tract, leading to severe acute respiratory syndrome and potential mortality. Despite the approval of numerous vaccines, they have not been entirely effective in halting the life-threatening pandemic^2^. This could be attributed to issues such as insufficient and unavailable vaccination supplies, the emergence of virus mutations, and a shortage of viable alternative therapeutic approaches^3^. The quickest and easiest solution was to employ FDA-approved medications like lopinavir/ritonavir, remdesivir, and favipiravir because the process of approving a novel treatment for human use is drawn out and entails several steps^4^.

**Fig. 1:**
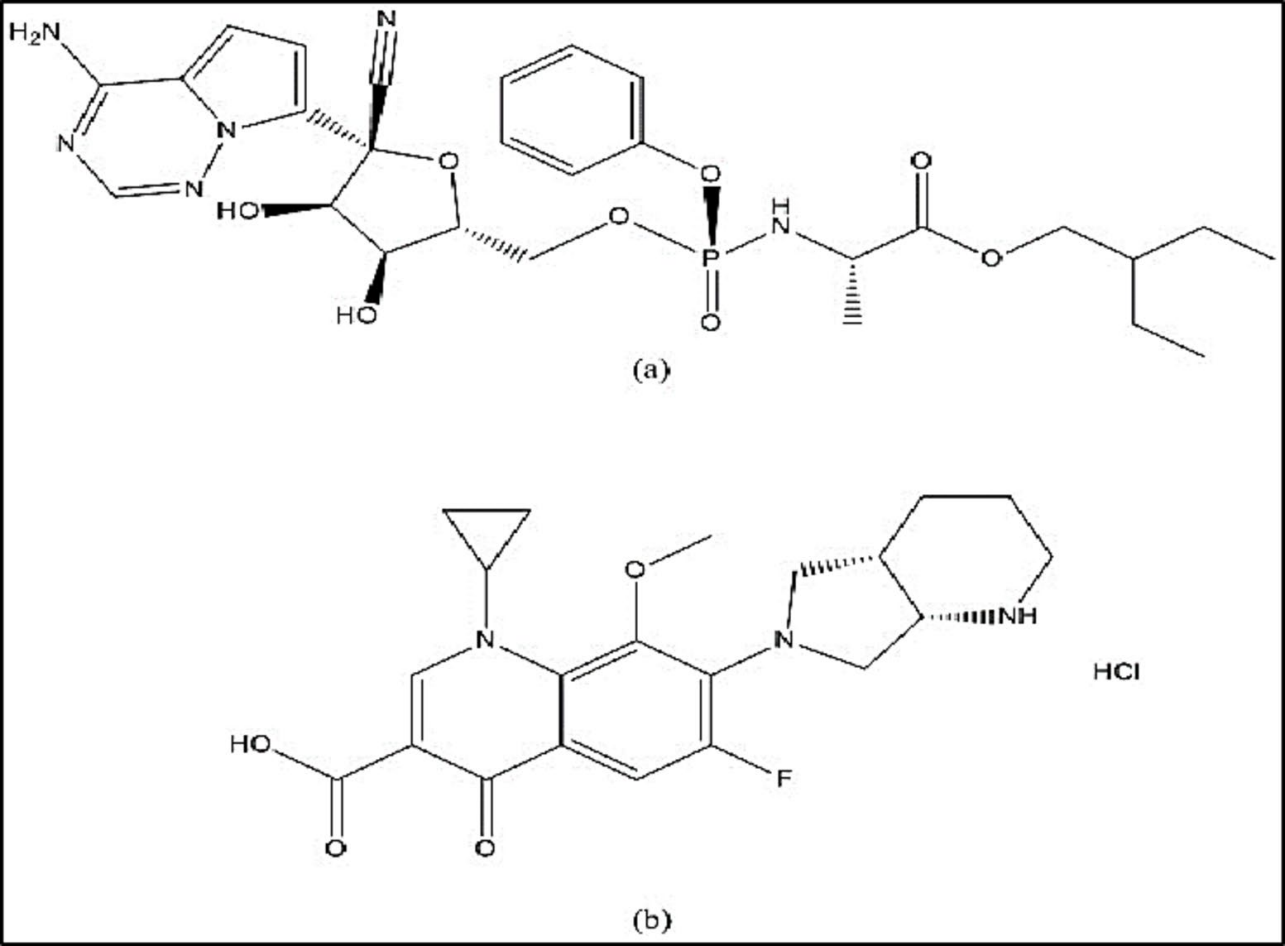
RDV (**a**) and MFX (**b**) chemical structures.

Remdesivir (RDV), (Fig. 1a), chemically named (2-ethylbutyl (2 S)-2-[[[(2R,3 S,4R,5R)-5-(4-aminopyrrolo[2,1-f][1,2,4]triazin-7-yl)-5-cyano-3,4-dihydroxyoxolan-2-yl]methoxy-phenoxy-phosphoryl]amino]propanoate) is an adenosine triphosphate analog that has wide antiviral action. RDV, was designed by Gilead Sciences to treat Ebola virus infection and has been approved through the FDA to treat SARS-CoV-2^5,6^. RDV works by attaching itself to viral RNA polymerase and halts RNA transcription prematurely to prevent viral reproduction^7^. It has been reported that RDV can be estimated by chromatographic^8–11^, spectrofluorimetric^12–16^, spectrophotometric^17–22^, and electrochemical techniques^23,24^, in different matrices.

While COVID-19 is caused by a viral infection, the frequent utilization of antibacterial agents for its treatment raises concerns. Antibiotics do not directly impact SARS-CoV-2, but viral respiratory infections can make individuals more susceptible to bacterial pneumonia^25^. Fluoroquinolones such as moxifloxacin hydrochloride (MFX), Fig. 1b, chemically named (7-[(4aS,7aS)-1,2,3,4,4a,5,7,7a-octahydropyrrolo[3,4-b]pyridin-6-yl]-1-cyclopropyl-6-fluoro-8-me-thoxy-4-oxoquinoline-3-carboxylic acid; hydrochloride), have notable immunomodulatory effects, including downregulating IL-1 and TNF, and have been shown to possess antiviral characteristics^26^. For this reason, these compounds might be helpful in treating COVID-19 infection and associated respiratory issues^27^. The literature review indicates a number of analytical methods for MFX analysis, including spectrophotometric^28–31^ ,liquid chromatographic^32–37^ spectrofluorometric^38–41^ and densitometric methods^42,43^. It is noteworthy that the respiratory fluoroquinolones as MFX are the first line of treatment for severe community-acquired pneumonia. They have comparable safety profiles to other antibiotics used to treat respiratory infections, such as macrolides and β-lactams. They also have favorable pharmacokinetic features and greater lung concentrations^44,45^. Because MFX is used as an add-on therapy for COVID-19, it is important to measure the levels of RDV and MFX in human plasma^45^. With respect to the pharmacokinetic profile of RDV and MFX, their corresponding Cmax values of 4420 ng/mL and 3.56 µg/mL^46,47^ are sufficiently high to be determined by spectrophotometry in the plasma matrix.

No existing method has been reported for the simultaneous analysis of RDV and MFX in co-formulations and biological fluids as co-administered drugs. This highlights the need for a method that can monitor these medications concurrently, particularly for hospitalized patients requiring therapeutic drug monitoring. The goal of this study is to develop the first analytical method capable of quantifying these co-administered drugs in both co-formulations and human plasma, with a focus on green analytical chemistry principles.

Consequently, it is the first developed green spectrophotometric methodologies to estimate RDV and MFX in different matrices. It is challenging to resolve RDV and MFX UV absorption spectra directly in mixtures due to their significant overlap. Therefore, we present in this paper three simple and sensitive spectrophotometric extension area-based techniques^48,49^ for the selective simultaneous quantitative detection of RDV and MFX without prior separation. The proposed approaches were absorbance subtraction (AS), extended ratio subtraction (EXRS) and amplitude modulation (AM) methods.

Since 1999, the phrase “green analytical chemistry” has been proposed for discussion. The majority of measures aimed at making chemical procedures more environmentally friendly emphasize cutting back on reagents, using less energy, and using more safe solvents^50^. The ecoscale penalty points approach, and the green analytical procedure index (GAPI) were utilized to evaluate the greenness of the suggested procedures, and all developed methods were thoroughly validated^50,51^.

## Experimental

### Materials and reagents

EVA Pharma for Pharmaceuticals and Medical Appliances (Cairo, Egypt) generously donated the powders of remdesivir (99.15%) and MFX (99.45%). Remdesivir-Evapharma^®^ vials containing 100 mg of RDV, manufactured by EVA Pharma (Cairo, Egypt), were acquired from the local market (batch numbers: 2009561, 2009563, 2009566, 2009567). Moxiflox^®^ pills containing 400 mg moxifloxacin per tablet, manufactured by EVA Pharma (Cairo, Egypt), were also purchased locally (batch number 2212537).

### Solvents

Methanol, analytical grade (Sigma–Aldrich, Germany).

### Instruments

The laboratory employed a Shimadzu UV-Visible 1800 Spectrophotometer (Tokyo, Japan) with matching 10 mm quartz cells. Version 2.21 of Shimadzu’s UV-Probe personal spectroscopy software was used to run the equipment. Additionally, an analytical balance (Precisa125A, Switzerland) was used in the studies.

### Standard solutions

In the preparation of RDV and MFX stock standards, 100 mg of each drug powder was dissolved in 70 mL of methanol. The solutions were sonicated for 15 min, and then the volume was adjusted to 100 mL with methanol. The stock solutions were then diluted with methanol to obtain working standard solutions of 100 µg/mL.

### Laboratory prepared mixtures

The standard solutions of RDV and MFX were accurately aliquoted into 10 mL volumetric flasks, and methanol was used to dilute the solution with various ratios.

### Procedures

#### Calibration curves construction

Two sets of 10-mL volumetric flasks were filled with aliquots of RDV and MFX standard solutions (100 µg/mL) ranging from (10–150) µg and (10–100) µg, respectively. Dilute to the end with methanol. In the 200–400 nm range we measured the absorbance of each pair at the zero order using methanol as the blank.

#### Absorbance subtraction method

This innovative technique uses a straightforward factor that is computed theoretically to enable quantitative estimate of both X and Y in their binary combination (X + Y) using a single regression equation. Analyzing the absorbance values of zero-order spectra at the isoabsorptive point versus the concentrations of X and Y produces regression equations^49,52,53^

RDV absorbance values at 229 nm (λ_iso_) were measured, and a calibration graph was created to link the absorbance to the drug concentration in µg/mL. MFX absorbance values at 229 and 360 nm were measured, and the absorbance factor [A229/A360] was computed. To find the absorbance of MFX in combination with RDV at 240 nm, the absorbance at 360 nm of the combination was multiplied by its absorbance factor. Ultimately, the absorbance of RDV at 229 nm was calculated by deducting the absorbance of MFX from the mixture’s absorbance at this wavelength. Each drug concentration at 229 nm can be estimated using the isoabsorptive point regression equation.

#### Extended ratio subtraction method

To find the components of X and Y, one divides the spectrum of a mixture by a concentration of Y, as a divisor (Y’). This leads to the creation of a new curve that shows X/Y’ + (Y/Y’) = (X/Y’) + Constant. After that, the original curve of X can be retrieved by deducting the constant from it then multiplication by (Y'). By dividing the obtained original curve of X by a known concentration of X as a divisor (X’), one can find the value of constant X/X’. Dividing the spectrum of the mixture (X + Y) by the same divisor X’, the division will give a new curve that represents (X/X’) + (Y/X’) where X/X’ is the previously obtained constant. If we subtract this constant, then multiply the obtained curve after subtraction by X’ (the divisor), therefore we can obtain the original curve of Y.

In order to compute X and Y concentrations, regression equations that depict the linear relationship between absorbance at λ_max_ and concentrations can be used^52,54^.

Our ratio spectrum was calculated by dividing the combination of medications by the spectrum of MFX solution (6 µg/mL). Each ratio spectrum had its constant (the amplitude value at 360 nm) subtracted from it, and the resulting spectra were then multiplied by the divisor spectrum (MFX solution (6 µg/mL)). RDV’s amplitude values were measured at 245 nm, and a calibration curve was made to link these values to the relevant concentrations of RDV in µg/mL.

In the case of MFX, a new ratio spectrum with a constant amplitude value, particularly at 245 nm, was created by dividing the obtained RDV absorption spectrum by the spectrum of RDV solution (8 µg/mL). In order to identify the ratio spectrum, we divided the absorption spectrum of the combination drug by the spectrum of the RDV solution (8 µg/mL). Each ratio spectrum had the constant (amplitude value at 245 nm in the ratio spectra) subtracted from it. The resulting spectra were then multiplied by the divisor spectrum [remdesivir solution (8 µg/mL)]. Based on the amplitude measurements at 295 nm for MFX, a calibration graph was developed to correlate these values with moxifloxacin concentrations in µg/mL.

#### Amplitude modulation method

A combination of X + Y, with Y being more extended than X, is used in the AM approach, which manipulates ratio spectrum techniques. The isosbestic point of the ratio spectrum can be determined by dividing the mixture spectrum by Y’s spectrum. With the help of a unified regression equation, the peak amplitude at the extended part can be correlated with Y concentration^49^. In order to determine RDV’s ratio spectrum, we divided its absorption spectrum by that of moxifloxacin (1 µg/mL). Next, a calibration graph for RDV was created through linking the concentrations of RDV in µg/mL to the amplitudes of the ratio spectra at 229 nm (λ_iso_). By dividing the regression equation at 229 nm by the normalized spectrum of MFX and subtracting the amplitude values at 360 nm, the concentration of RDV was determined. The ratio spectrum of MFX was obtained by dividing its absorption spectrum by the normalized absorption spectrum of the compound (1 µg/mL). Next, a calibration graph was created for MFX, which related the concentrations of the drug in µg/mL to the amplitudes of the ratio spectra at 360 nm (λ_max_). MFX concentration was determined by dividing the regression equation at 360 nm by the drug’s normalized spectrum.

### Methods applications

#### Laboratory prepared mixtures analysis

Spectra of synthetic laboratory-prepared mixtures were measured and processed in accordance with the suggested procedures following the preparation of various ratios of these mixtures.

#### Pharmaceutical formulation analysis

*RDV* Four vials of Remdesivir-Evapharma ^®^ (100 mg / 20 mL) were properly mixed. Two milliliters of RDV (10 mg) were accurately put in a 100-milliliter volumetric flask, and approximately 70 milliliters of methanol was added. The solution was vigorously shaken for 15 min before sonicating for 30 min. Methanol was added to a volume of 100 mL to reach a concentration of 100 µg/mL.

*MFX* Ten Moxiflox^®^ tablets (436.37 mg MFX equivalent to 400 mg moxifloxacin/tablet) were weighed and coarsely powdered. Accurately weighing the powder (17 mg) that equates to 10 mg of MFX, it was then transferred to a 100 mL volumetric flask and the amount was increased to roughly 70 mL using methanol. The solution was vigorously shaken for 15 min before sonicating for 30 min. Methanol was added to a volume of 100 mL to reach a concentration of 100 µg/mL.

*Remdesivir and moxifloxacin hydrochloride (co-formulated)* RDV and MFX fixed-dose pills were unavailable in Egypt, so a fixed-dose combination was developed. Four Remdesivir^®^ vials (100 mg/20 mL vial) and four finely powdered Moxiflox^®^ tablets (400 mg/tablet) were thoroughly combined. Two mL of this mixture (10 mg of RDV and 40 mg of MFX) was accurately put in a 100-mL volumetric flask, which was then filled with methanol to a volume of approximately 70 mL. The solution was vigorously shaken for 15 min before sonicating for 30 min. The volume was completed to 100 mL with methanol and then filtered to reach a concentration of (100 µg for RDV and 400 µg for MFX).

### The reported method

The reported method for RDV analysis includes two green UV spectrophotometric methods: ratio difference and first derivative of ratio spectra^22^.

UV spectrophotometric techniques were described for quantifying MFX in various medicinal formulations. The moxifloxacin concentration was measured at 289 nm in phosphate buffer (pH 7.4) and 296 nm in 0.1 N hydrochloric acid (pH 1.2)^31^.

### Spiked human plasma procedure

Drug-free plasma (1 mL) was centrifuged with aliquots of RDV and MFX standard solutions (100 μg/mL).To precipitate the proteins, 3 mL of methanol was added. Following a vortex shaker mix, the tubes were centrifuged for 30 min at 4000 rpm. The resulting supernatants, free of proteins, were evaporated to dryness under vacuum using a rotary evaporator. After dissolving the dry residue in methanol, it was put in 10-mL volumetric flasks and filled to the top with methanol. The general procedure was repeated for each drug at various concentrations within the working range. Both the MFX and the RDV contents have been estimated by corresponding regression analysis.

## Results and discussion

### Optimization of the methods

In this current study, three simple and sensitive spectrophotometric extension area-based techniques were proposed for the selective simultaneous quantitative detection of RDV and MFX without prior separation.

**Fig. 2:**
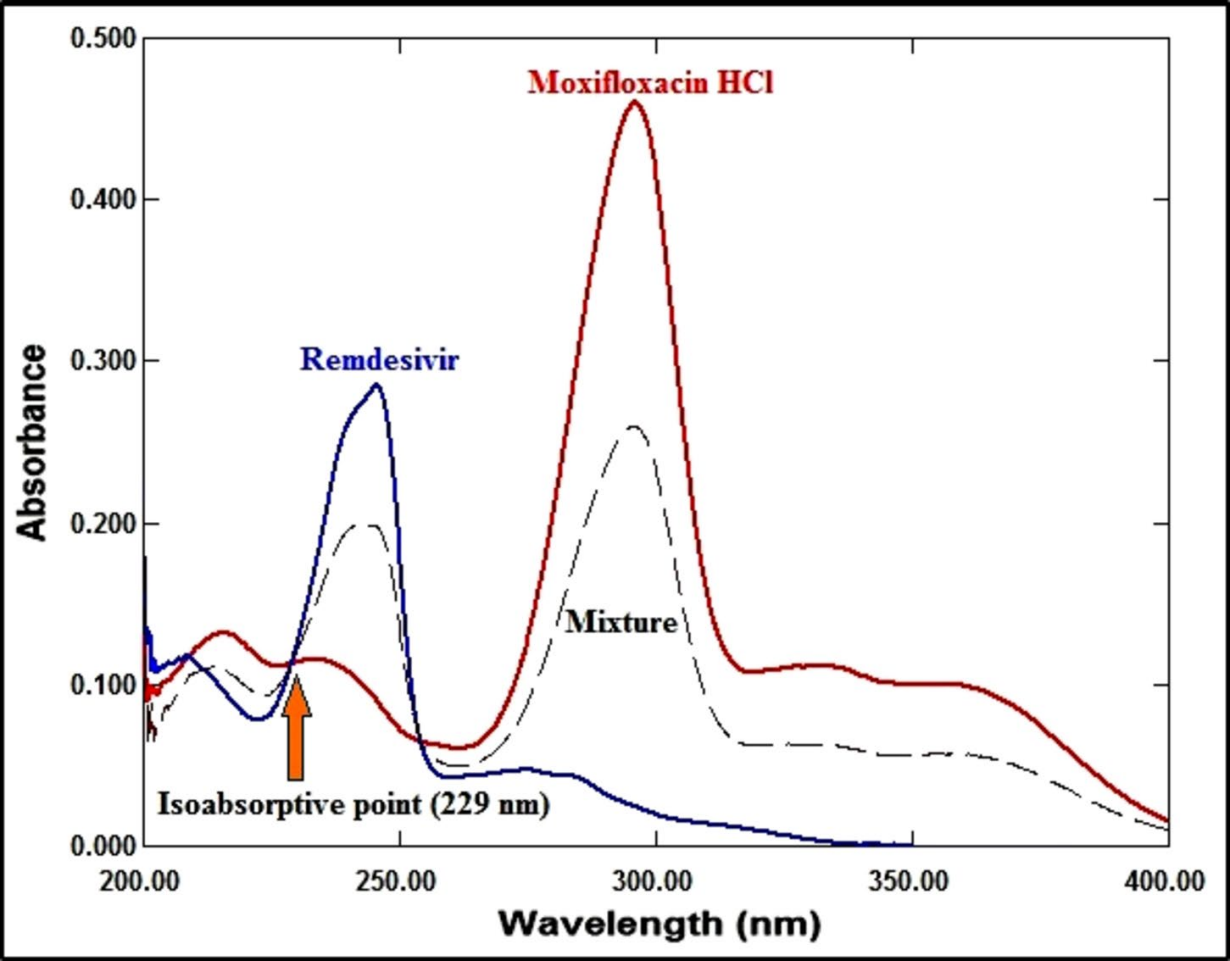
The absorption spectra of RDV (4 µg/mL), MFX (4 µg/mL), and a combination comprising 2 µg/mL of each medication in methanol measured at zero order.

*Spectral characteristics* As seen in Fig. 2, the zero-order absorption spectra of RDV and MFX exhibit significant overlap, with an isoabsorptive point at 229 nm for the former and a more extended plateau region from 340 to 400 nm for the latter, where RDV has no absorbance.

#### Absorbance subtraction method

The absorbance factor (A229/A360) for MFX was estimated by measuring various concentrations and taking the average, which was found to be 1.128. MFX absorbance at 229 nm in a combination containing RDV can be calculated by multiplying its absorbance at 360 nm by the absorbance factor. To calculate RDV absorbance at 229 nm, subtract the absorbance of MFX at 229 nm from the total absorbance of the mixture at the same wavelength. Each drug’s concentration could be determined using the isoabsorptive point regression equation at 229 nm.

#### Extended ratio subtraction method

**Fig. 3:**
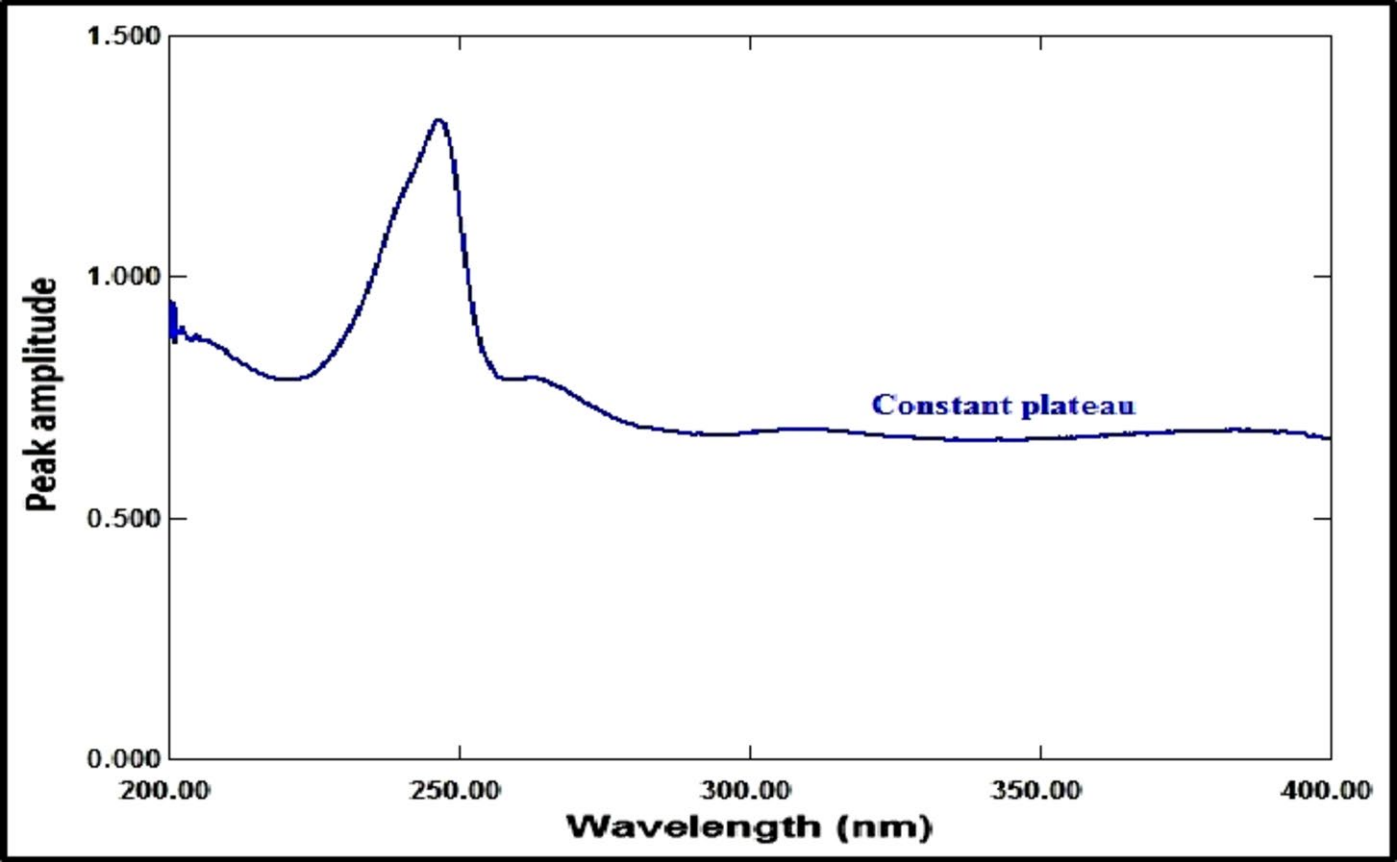
Ratio spectrum of laboratory prepared mixture of RDV (1 µg/mL) and MFX (4 µg/mL) using 6 µg/mL of MFX as a divisor.

**Fig. 4:**
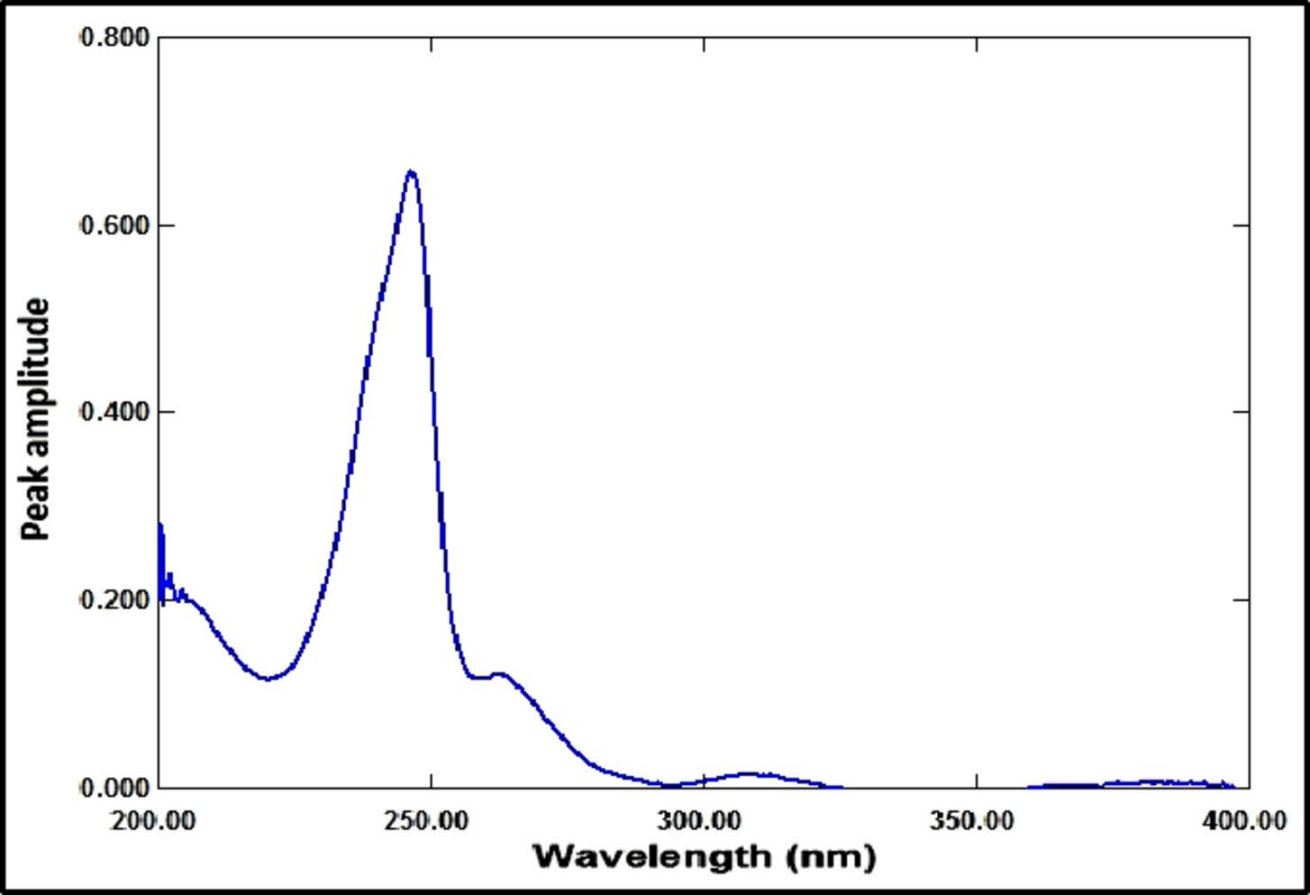
Ratio spectrum of a mixture of RDV (1 µg/mL) and MFX (4 µg/mL) made in a laboratory. After subtracting the constant at 360 nm, 6 µg/mL of MFX was used as a divisor.

**Fig. 5:**
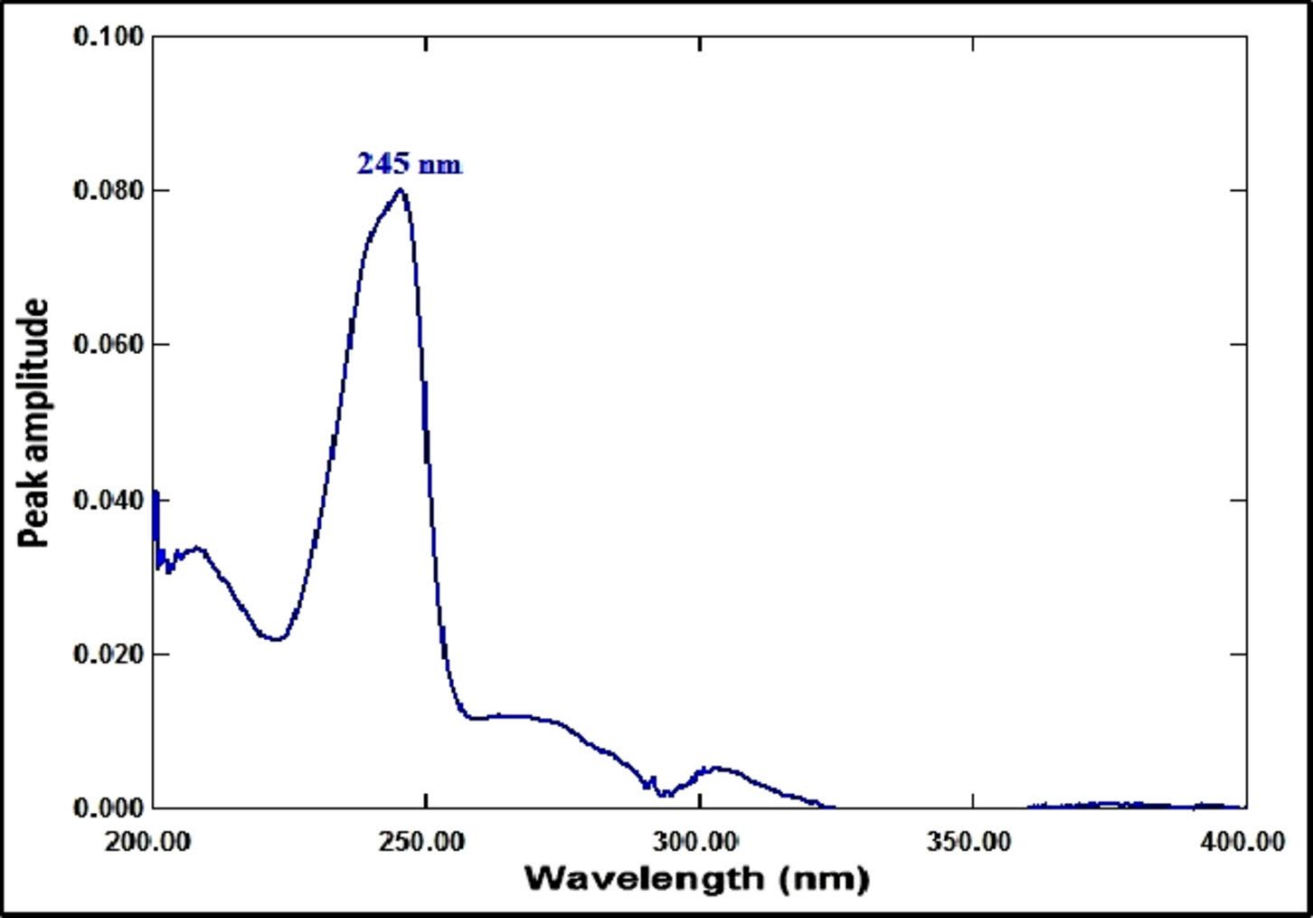
The ultimate RDV (1 µg/mL) spectrum acquired subsequent to divisor multiplication (6 µg/mL of MFX).

**Fig. 6:**
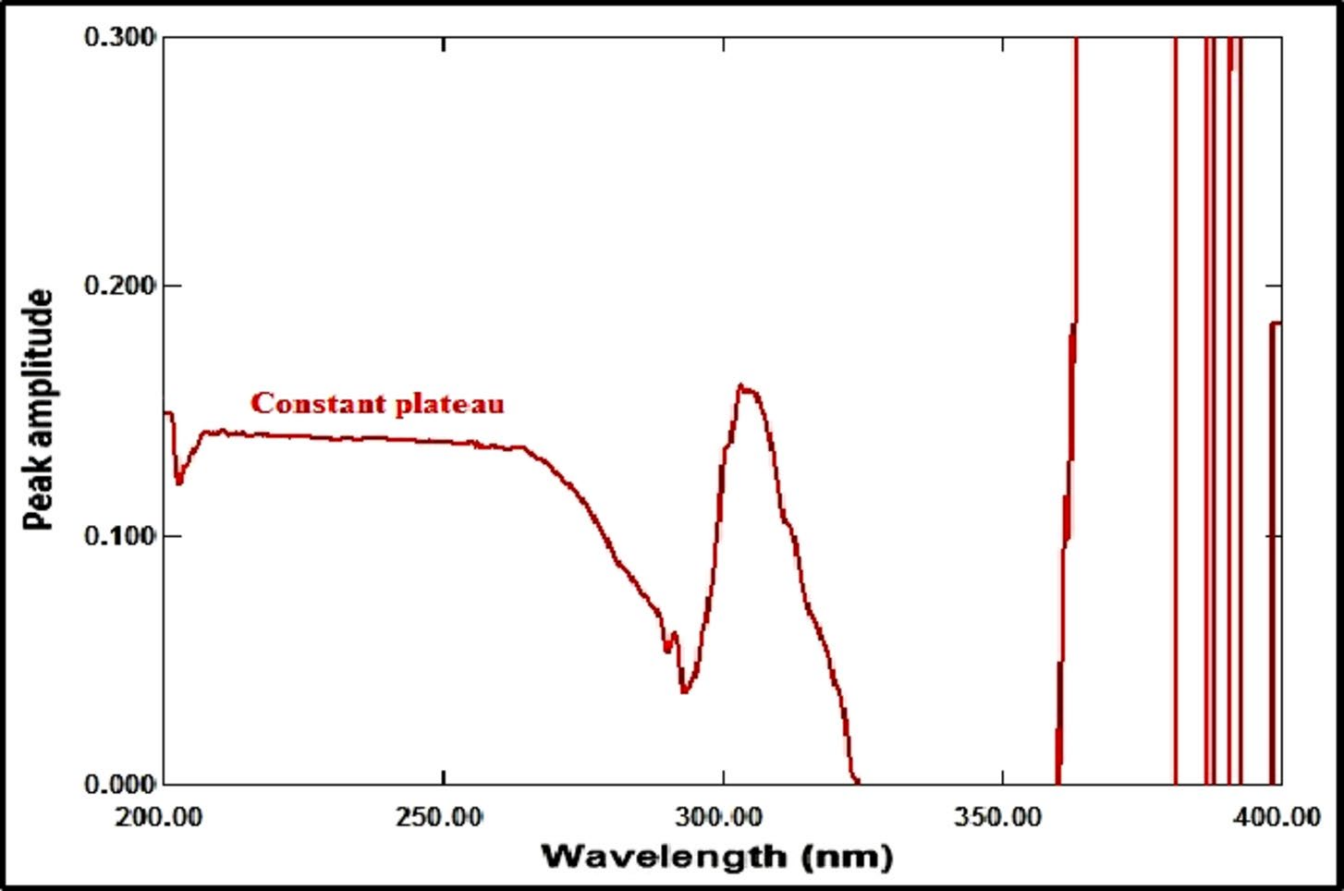
Ratio spectrum derived from dividing the RDV final spectrum (1 µg/mL) by the RDV divisor (8 µg/mL).

**Fig. 7:**
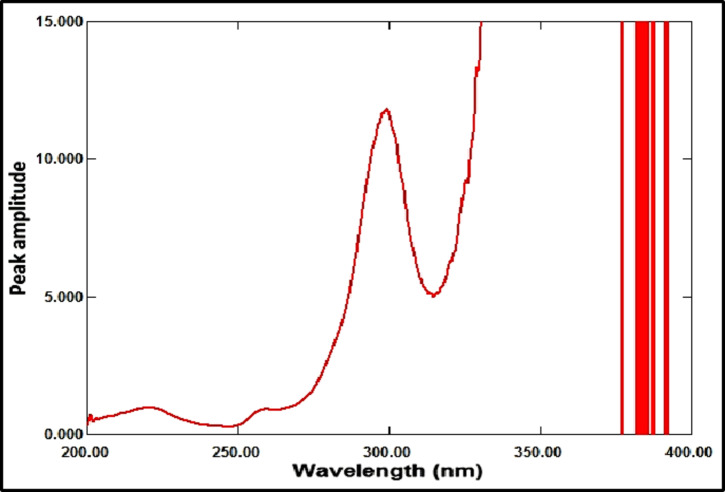
Ratio spectrum of a mixture of MFX (4 µg/mL) and RDV (1 µg/mL) made in a laboratory using 8 µg/mL of RDV as a divisor.

**Fig. 8:**
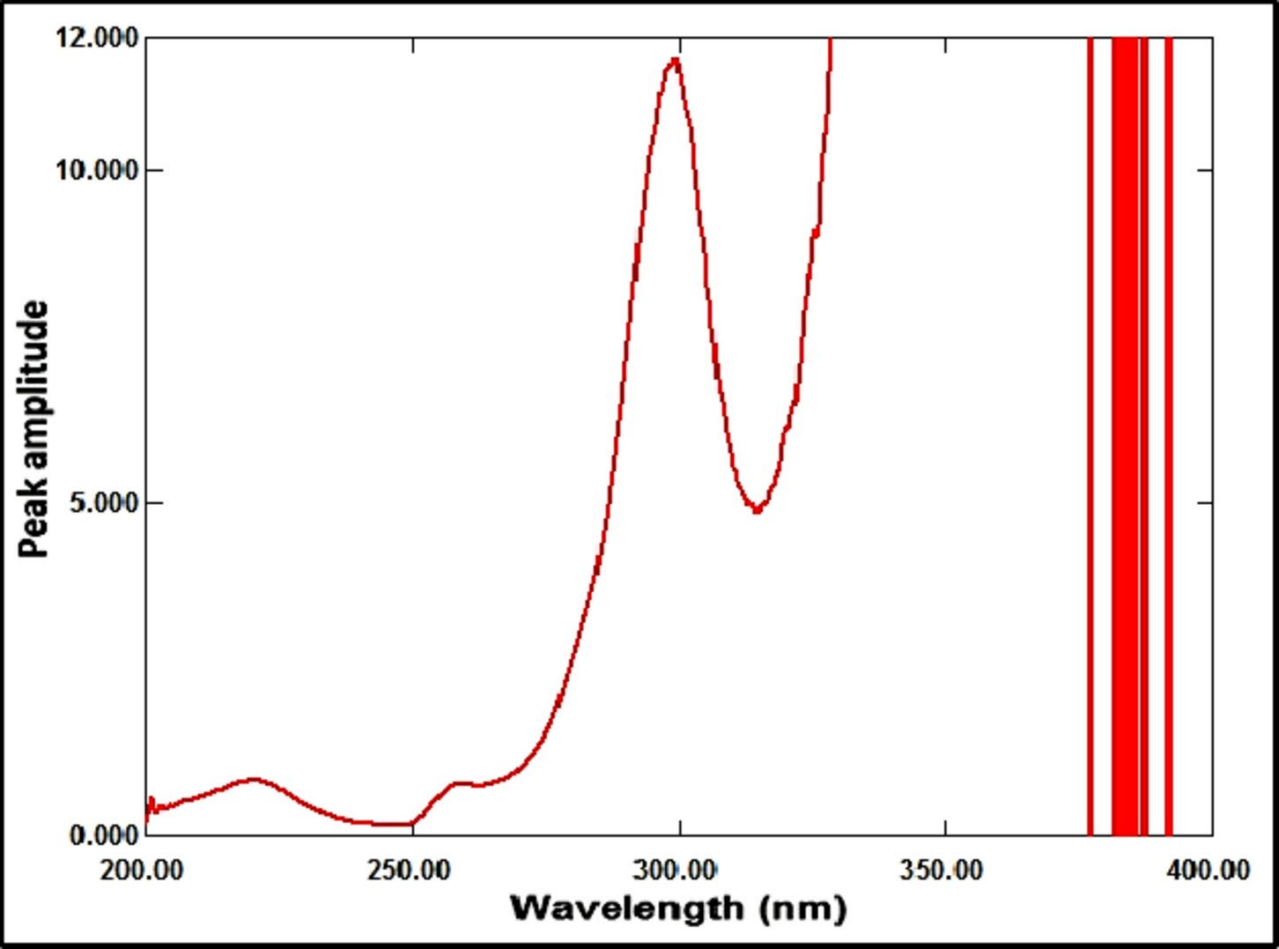
Ratio spectrum of a mixture of MFX (4 µg/mL) and RDV (1 µg/mL) made in a laboratory, using RDV (8 µg/mL) as a divisor after the constant at 245 nm was subtracted.

**Fig. 9:**
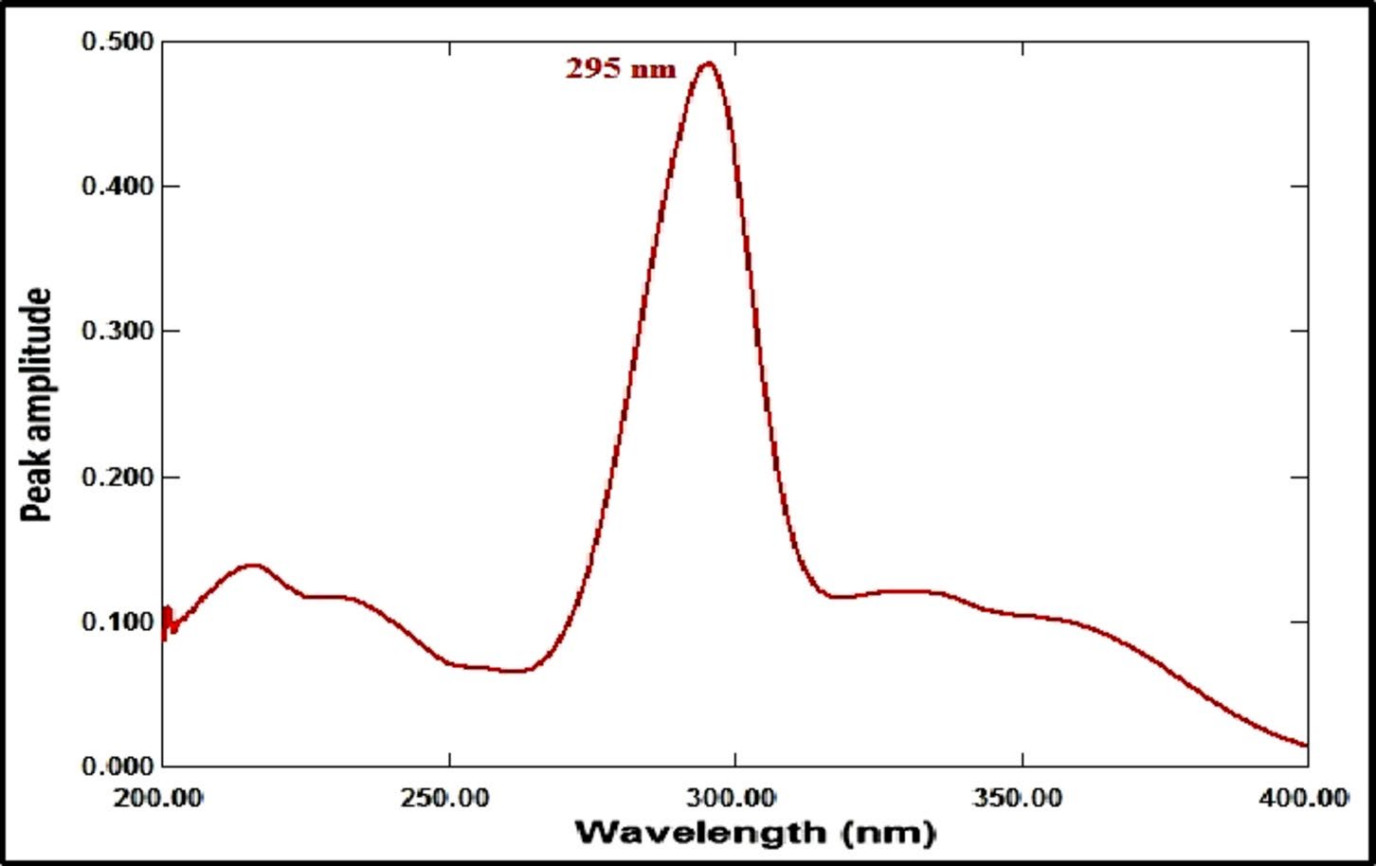
The MFX (1 µg/mL) final spectrum, acquired via divisor multiplication (8 µg/mL of RDV).

The ratio spectrum was produced by using the absorption spectrum of MFX (6 µg/mL) as a divisor and dividing the absorption spectra of the mixtures of RDV and MFX by this divisor, as shown in Fig. 3. Figure 4 shows the absorbance values in the plateau region at 360 nm were subtracted, and the results were multiplied by the spectrum of the divider, MFX (6 µg /mL)], as indicated in Fig. 5. The peak amplitude at 245 nm in the final RDV spectrum is proportional to the RDV concentration, with no interference from MFX. For MFX, the absorption spectra of RDV was divided by the spectrum of RDV solution (8 µg/mL), resulting in a new ratio spectrum with a consistent amplitude value, particularly at 245 nm (Fig. 6). For the ratio spectra shown in Fig. 7, the absorption spectra of mixed medications were divided by the spectrum of RDV solution (8 µg/mL). Figure 8 shows that the constant (amplitude value at 245 nm in the ratio spectra) was subtracted from each ratio spectrum. The resultant spectra were then multiplied by the divisor spectrum [RDV solution (8 µg/mL), as shown in Fig. 9. The peak amplitude at 295 nm in the final MFX spectrum is proportional to the MFX concentration, without interference from RDV. The divisor concentration has to be carefully chosen; thus, numerous trials were conducted utilizing various divisor concentrations.

#### Amplitude modulation method

**Fig. 10:**
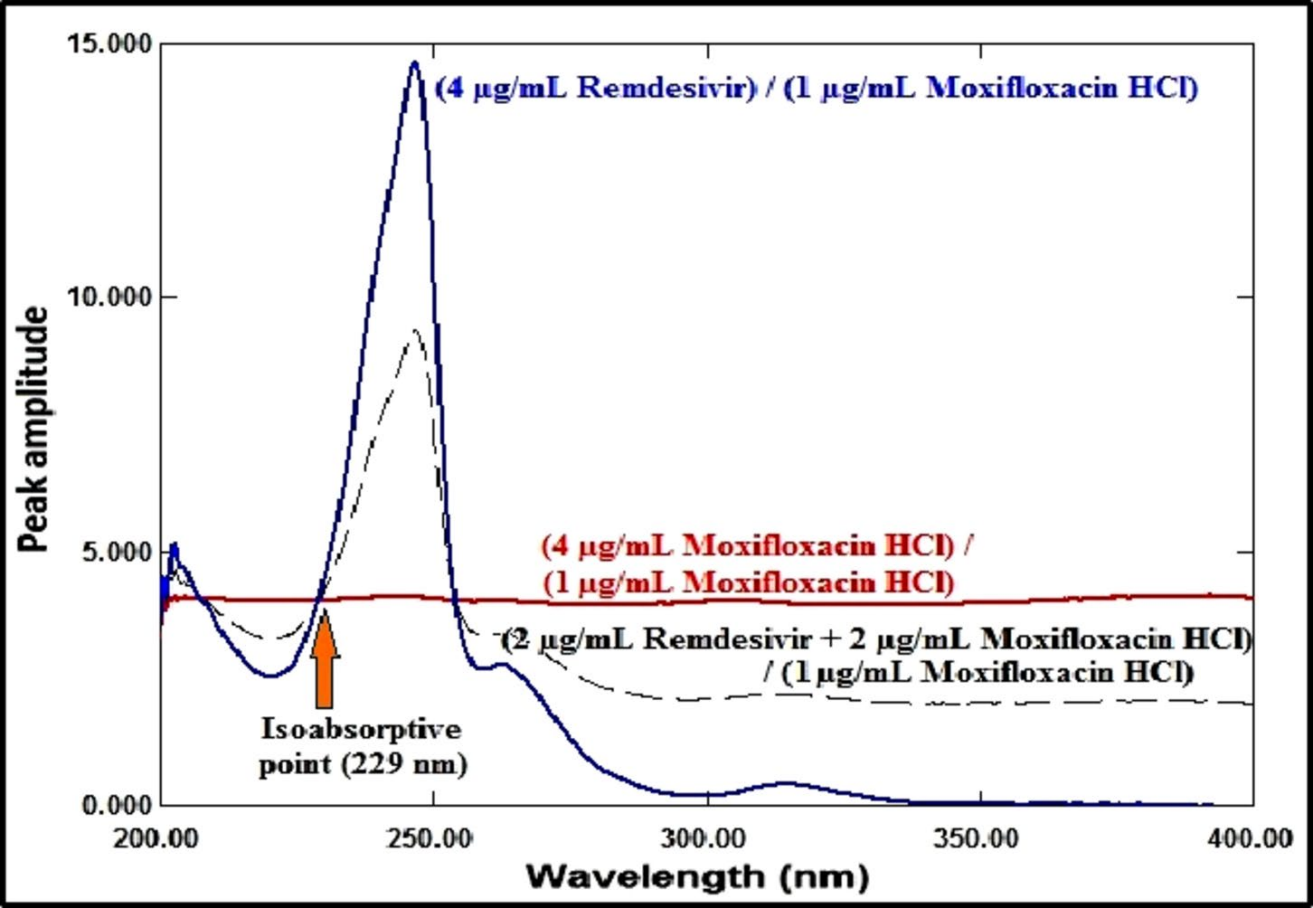
Ratio spectra of RDV (4 µg/mL), MFX (4 µg/mL), and mixture of 2 µg/mL of each drug using MFX (1 µg/mL) normalized spectrum as a divisor.

**Fig. 11:**
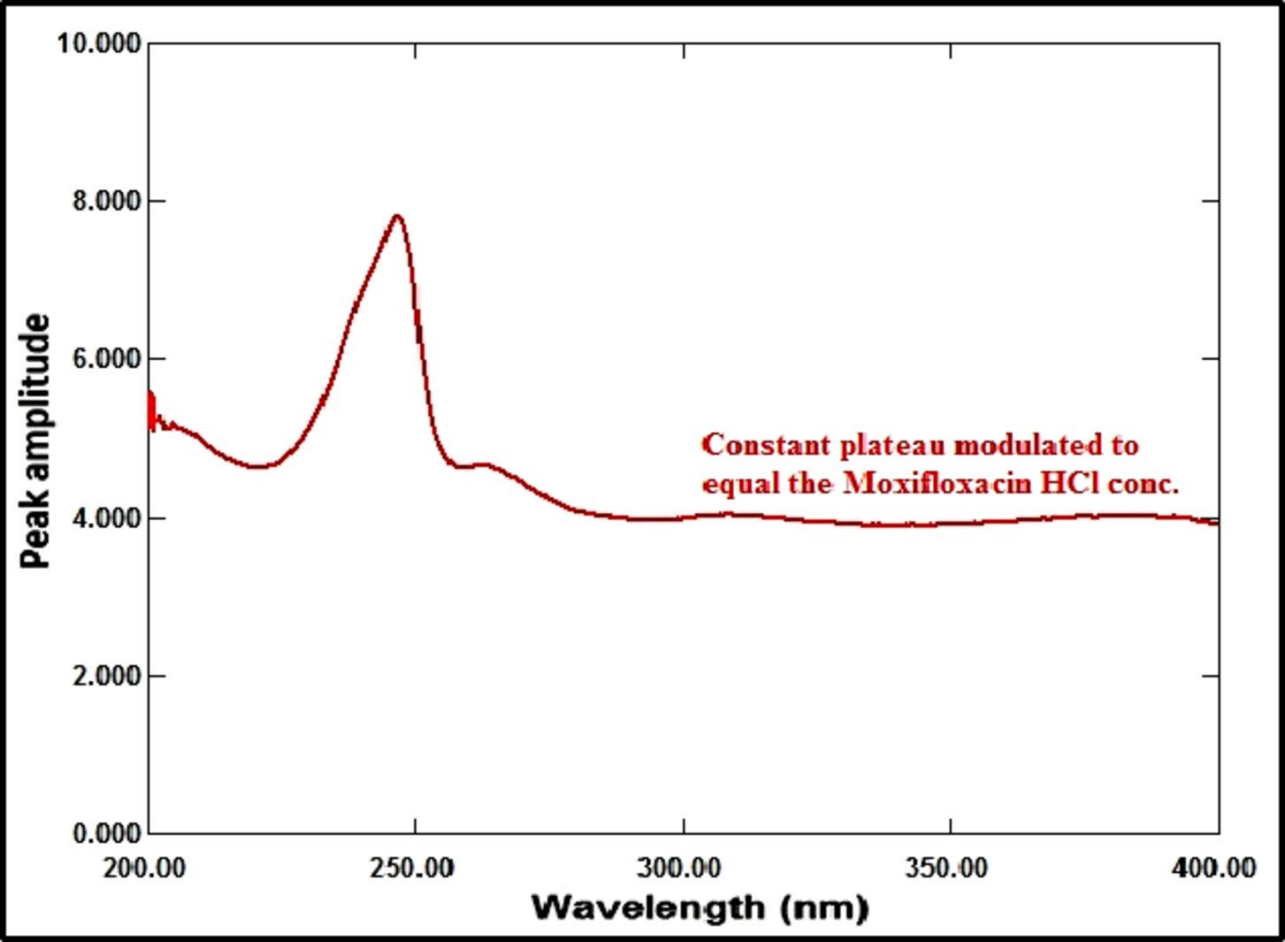
Ratio spectra of laboratory prepared mixtures of RDV (1 µg/mL) and MFX (4 µg/mL) using normalized spectrum of MFX (1 µg/mL) as a divisor.

**Fig. 12:**
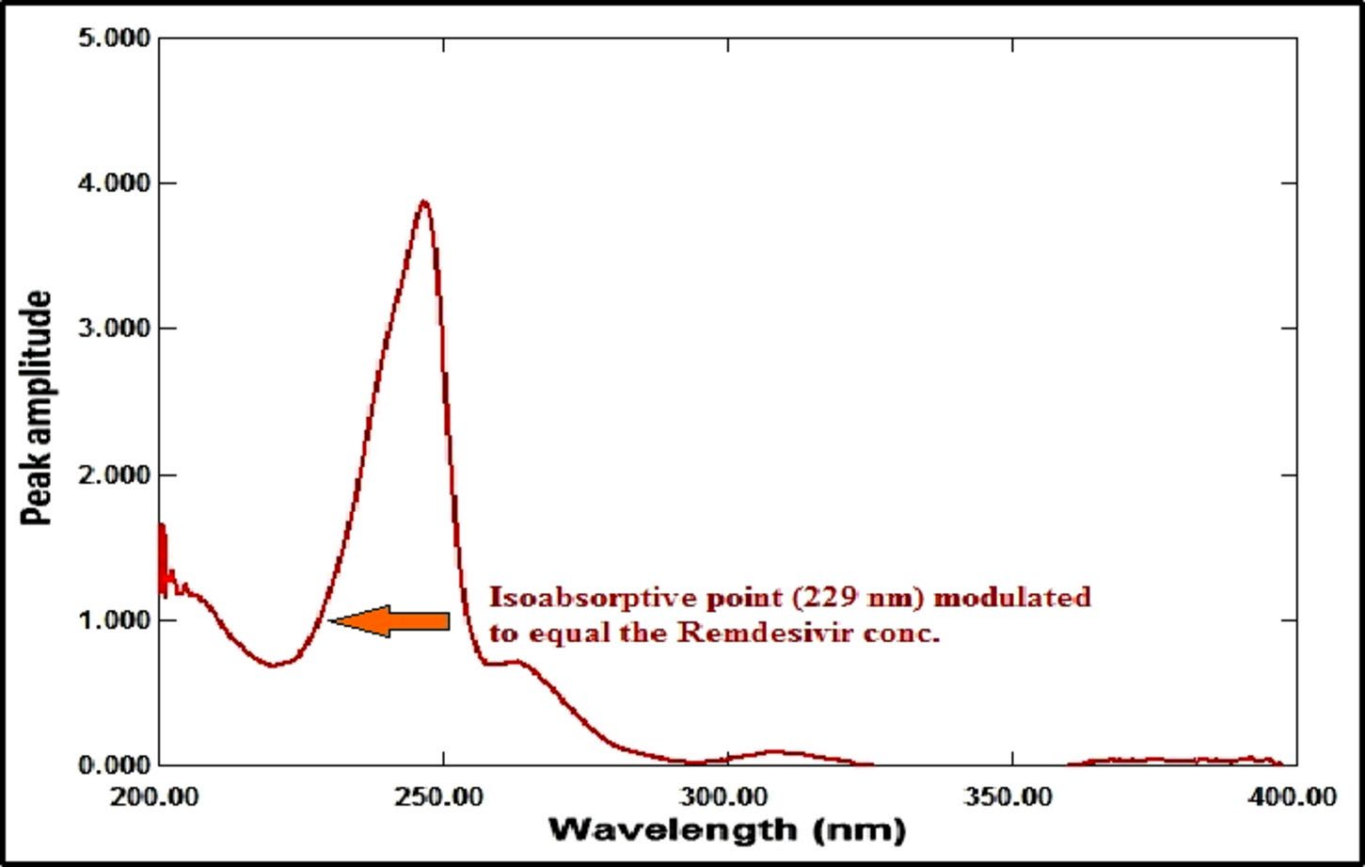
Ratio spectra of laboratory prepared mixtures of RDV (1 µg/mL) and MFX (4 µg/mL) using normalized spectrum of MFX (1 µg/mL) as a divisor after subtraction of the constant at 360 nm.

RDV and MFX’s zero-order absorption spectra display an isoabsorptive point at 229 nm, which is maintained at the ratio spectra as illustrated in Fig. 10. The binary mixture’s spectrum is divided by the normalized MFX spectrum (1 µg/mL) to generate the ratio spectra, as shown in Fig. 11. The MFX amplitude constant value can be found immediately at the plateau area at 360 nm, which is the constant’s amplitude value across the spectrum. The ratio spectrum plateau region at 360 nm has an amplitude value that is proportional to the concentration of MFX. At the isoabsorptive point (229 nm), the amplitude of the ratio spectra will be modified to match the total of the amplitudes of RDV and MFX. Subtracting the amplitude values in the plateau region at 360 nm (the constant), as shown in Fig. 12, yields the corresponding recorded amplitude of RDV at 229 nm, which corresponds to the recorded concentration of RDV in the mixture. RDV’s true concentration can be ascertained using its regression equation at the isoabsorptive point of 229 nm, avoiding mistakes brought on the signal-to-noise ratio. Likewise, the true concentration of MFX might be ascertained by using its regression equation in the plateau area, or 360 nm.

### Method validation

The suggested techniques were successfully validated and produced satisfactory results in accordance with ICH criteria^55^.

#### Linearity and range

Plotting the absorbance of RDV at 229 nm, the amplitudes of the final spectra at 245 nm, or the amplitudes of the ratio spectra at 229 nm for AS, EXRS, and AM methods, respectively, against the drug concentrations in µg/mL allowed to construct of the calibration graphs for the three methods within the parameters of the experiment that have been presented. For MFX, the calibration graphs were created by graphing the concentration of the drug in µg/mL against the absorbance of the compound at 229 nm, the amplitudes of the final spectra at 295 nm, or the plateau region of the ratio spectra at 360 nm for the AS, EXRS, and AM methods, respectively. It was discovered that the regression plots for RDV and MFX were linear over the range of 1–15 µg/mL and 1–10 µg/mL, respectively. Table 1 displayed the regression data and the calibration graphs’ strong linearity was demonstrated by the coefficient of determination values.

#### Detection and quantitation Limits

Calculating LODs and LOQs values, the results revealed the analytical sensitivity of the suggested methodologies for the medicines under study, as presented in Table 1.

**Table 1:** Regression and validation data for determining RDV and MFX using the proposed methods.

Parameters	RDV			MFX		
	AS	EXRS	AM	AS	EXRS	AM
Wavelength (nm)	229	245	229	229	295	360
Linearity range (µg/mL)	1–5	1–15	1–15	1–10	1–10	1–10
Slope	0.0275	0.0708	0.9493	0.0293	0.1219	0.9933
Intercept	0.0054	0.0089	0.1337	-0.0011	-0.0020	-0.0167
LOD (µg/mL)	0.301	0.278	0.296	0.273	0.259	0.187
LOQ (µg/mL)	0.911	0.844	0.897	0.828	0.785	0.567
Coefficient of determination (r^2^)	0.9995	0.9997	0.9997	0.9997	0.9996	0.9996
Accuracy (% R)^a^	99.25	100.94	98.95	98.70	99.23	100.35
Precision (% RSD)^b^						
Repeatability	1.165	0.762	1.021	1.019	0.987	0.883
Intermediate precision	1.113	1.540	0.635	1.448	1.237	1.549

^a^Average of 9 determinations (3 concentrations repeated 3 times).

^b^%RSD of 9 determinations (3 concentrations repeated 3 times).

#### Accuracy and precision

RDV’s (3, 9 and 12 µg/mL) and MFX’s (2, 5 and 8 µg/mL) concentration levels covering the linearity range of the drugs under investigation were determined in triplicate using the procedure described, and the accuracy of the methods was measured as the mean percent recovery (%R). The accuracy of the suggested approaches was demonstrated by the findings in Table 1. For repeatability, three concentration levels covering each drug’s linearity range 3, 9, and 12 µg/mL for RDV and 2, 5, and 8 µg/mL for MFX were determined in triplicate in a single day, and for intermediate precision, over three consecutive days. This allowed for the calculation of the methods’ precision, expressed as a percent relative standard deviation (%RSD). %RSD results were low that indicated the method’s excellent precision as seen in Table 1.

#### Specificity

Synthetic mixtures with varying proportions of the medications under study were made and thoroughly mixed. The combinations were then examined utilizing the method’s previously outlined basic approach. Excellent, satisfactory outcomes were attained and are shown in Table 2. The suggested techniques were used to determine the single dosage form of RDV and the co-formulated dosage form of MFX in the laboratory, as shown in Table 3. To assess the impact of the matrix on the identification of the two medications, specificity was also assessed by using the conventional addition approach to pharmaceutical preparations that had already undergone analysis. Table 4 of the data showed that the suggested method was able to evaluate the medicine selectively and free from excipient interference.

**Table 2:** Analysis of laboratory prepared mixture of RDV and MFX by the proposed methods.

Method	Laboratory prepared mixture (µg/mL)		% Recovery*	
	RDV	MFX	RDV	MFX
AS	1	2	98.51	100.02
	1	4	98.90	101.96
	2	4	101.29	98.12
	2	8	100.56	99.57
	4	4	98.48	101.00
	Mean ± %RSD		99.55 ± 1.299	100.13 ± 1.457
EXRS	1	2	101.84	98.44
	1	4	100.42	99.88
	2	4	98.94	101.52
	2	8	99.65	100.90
	4	4	101.38	99.67
	Mean ± %RSD		100.44 ± 1.189	100.08 ± 1.186
AM	1	2	98.84	98.85
	1	4	99.37	99.69
	2	4	98.35	101.83
	2	8	99.19	100.82
	4	4	100.56	100.92
	Mean ± %RSD		99.26 ± 0.827	100.42 ± 1.157

*Average of 3 determinations.

**Table 3:** Determination of RDV and MFX using the suggested procedures in pharmaceutical formulations and co-formulated dosage forms.

Method	Remdesivir-Evapharma^®^ 100 mg/vial		Moxiflox^®^ 400 mg/tablet		Co-formulated dosage form			
					RDV		MFX	
	Conc. (µg/mL)	% R*	Conc. (µg/mL)	% R*	Conc. (µg/mL)	% R*	Conc. (µg/mL)	% R*
AS	1	96.73	4	98.21	1	100.76	4	98.12
	1.5	100.85	6	100.17	1.5	98.33	6	101.33
	2	99.27	8	99.45	2	98.51	8	100.05
	2.5	98.33	10	99.35	2.5	100.07	10	99.28
	Mean	98.79	Mean	99.29	Mean	99.42	Mean	99.70
	%RSD	1.747	%RSD	0.817	%RSD	1.195	%RSD	1.354
EXRS	1	101.84	4	100.90	1	99.01	4	100.49
	1.5	98.96	6	101.31	1.5	98.02	6	101.86
	2	101.06	8	100.80	2	101.77	8	99.36
	2.5	99.49	10	98.93	2.5	100.62	10	99.67
	Mean	100.34	Mean	100.49	Mean	99.86	Mean	100.35
	%RSD	1.333	%RSD	1.054	%RSD	1.667	%RSD	1.111
AM	1	101.05	4	100.79	1	101.05	4	100.99
	1.5	98.06	6	101.66	1.5	98.69	6	101.36
	2	98.56	8	99.14	2	98.04	8	99.24
	2.5	99.24	10	99.60	2.5	99.62	10	99.69
	Mean	99.23	Mean	100.30	Mean	99.35	Mean	100.32
	%RSD	1.320	%RSD	1.141	%RSD	1.317	%RSD	1.013

*Average of 3 determinations.

**Table 4:** Standard addition technique applied using suggested methods.

Pharmaceutical (µg/mL)	RDV				MFX			
	Pure added (µg/mL)	% R*			Pure added (µg/mL)	% R*		
		AS	EXRS	AM		AS	EXRS	AM
RDV 1 (1.01)* MFX 4 (3.93)*	1	96.73	97.60	98.10	3	99.09	98.17	99.66
	2	101.09	100.35	98.46	4	101.62	99.26	101.90
	3	98.91	98.92	100.68	5	100.41	101.07	98.21
Mean		98.91	98.96	99.08		100.37	99.50	99.92
%RSD		2.206	1.392	1.410		1.261	1.471	1.860

*Average of 3 determinations.

### Pharmaceutical applications

The concentration of RDV and MFX in a single dosage form or in a dosage form that was co-formulated in the lab was measured using the suggested methods. The standard addition process results corroborated the satisfactory findings, which were obtained in good accord with the label claim that revealed no signs of additives or excipients interference, as shown in Table 4. Statistics were used to compare the results with those attained by the previously mentioned methods^56–58^. There were no significant differences observed using the student’s t-test and F-test at the 95% confidence level^59^, showing that the proposed method for analyzing the tested substance in its pharmaceutical dose form is accurate and precise, as indicated in Table 5.

**Table 5:** Statistical analysis and determination of MFX and RDV in pharmaceutical preparations using reported and suggested spectrophotometric methods.

Parameters	RDV				MFX			
	AS	EXRS	AM	Reported method^22^	AS	EXRS	AM	Reported method^31^
Number of measurements	4	4	4	4	4	4	4	4
Mean % recovery	98.79	100.34	99.23	99.79	99.29	100.49	100.30	99.49
% RSD	1.747	1.333	1.320	1.197	0.817	1.054	1.141	1.496
Variance	2.980	1.790	1.715	1.428	0.658	1.122	1.310	2.216
Student’s *t*-test^a^ (2.447)	0.949	0.611	0.633	–	0.228	1.094	0.863	–
*F*-value^a^ (9.277)	2.087	1.253	1.201	–	3.368	1.975	1.691	–

^a^"t” and “F” tabulated values at (*P* = 0.05) are the values in parenthesis.

### Spiked human plasma analysis

Continuous monitoring of the medications in human plasma supports the idea that medication administration reduces mortality. The excellent sensitivity of the recently established methods, as indicated in Table 6, makes them suitable for use in the analysis of the medicines under investigation in human plasma.

**Table 6:** Determination of RDV and MFX in spiked human plasma using the described methods.

Method	RDV			MFX			
	Added (µg/mL)	Found* (µg/mL)	%Recovery	Added (µg/mL)	Found* (µg/mL)		%Recovery
AS	1	0.929	92.86	1	0.961		96.12
	1	0.953	95.34	4	3.771		94.27
	2	1.925	96.27	8	7.773		97.17
	3	2.849	94.97	4	3.732		93.30
	13	12.670	97.46	5	4.617		92.35
	Mean ± %RSD		95.38 ± 1.787	Mean ± %RSD			94.64 ± 2.096
EXRS	1	0.920	91.95	1	0.943		94.34
	1	0.948	94.77	4	3.880		97.01
	2	1.951	97.53	8	7.375		92.19
	3	2.883	96.09	4	3.741		93.52
	13	12.247	94.21	5	4.643		92.86
	Mean ± %RSD		94.91 ± 2.206	Mean ± %RSD			93.98 ± 1.988
AM	1	0.921	92.10	1		0.912	91.18
	1	0.952	95.15	4		3.882	97.043
	2	1.929	96.46	8		7.488	93.60
	3	2.841	94.71	4		3.625	90.62
	13	12.048	92.68	5		4.673	93.46
	Mean ± %RSD		94.22 ± 1.912	Mean ± %RSD			93.18 ± 2.720

*Average of 5 determinations.

### Assessment of greenness of the analytical methods

Using multiple greenness evaluation tools yields more accurate results and allows for a more comprehensive comparison of different analytical method^60^. Two greenness assessment metrics, the analytical eco-scale, and the green analytical procedure index (GAPI)^50,51^, were applied to assess the spectrophotometric techniques that were presented within the framework of green analytical chemistry. Furthermore, a comparison between the reported spectrophotometric methods and the specified method’s greenness was conducted. The analytical eco-scale is a semi-quantitative approach for assessing the environmental impact of analytical methodologies. The penalty points for the analytical procedure are determined based on two main parameters. The first parameter is the reagent parameter, which is calculated by evaluating the quantities, environmental impact, physical hazards, and health risks associated with the reagents used. The second parameter pertains to instrumentation, taking into account factors such as energy consumption, occupational hazards, and waste production. To calculate the greenness evaluation score, deduct the penalty points assigned to each criteria from 100. The green approach is classified as excellent or acceptable, or inadequate green based on its total score value. After calculating this score for the strategies we created and published, they were 91, 89 and 87 for proposed method, reported method for RDV and reported method for MFX, respectively, expressing excellent green characters.

The developed methodology, with a score of 91, is the most environmentally friendly spectrophotometric method when compared to other published methods. This is because it exclusively used methanol as a green solvent in plasma and for pharmaceutical applications. On the contrary, the reported method for RDV used ethanol as solvent and acetonitrile as extracting solvent in application of spiked plasma. The last reported method for MFX uses 0.1 N hydrochloric acid, phosphate buffer and acetone making it the least green method.

The GAPI approach, which is similarly depicted by a pictogram, is the second assessment method. There are fifteen parts in this colored pictogram. Depending on how green a certain segment is, it can be green, yellow, or red. The GAPI pictogram of the derivative spectrophotometric method demonstrates eight green segments, four yellow segments, and three red segments, while the GAPI pictogram of the RDV reported method shows seven green segments, five yellow segments, and three red segments. The other reported method (MFX) also exhibits seven green segments, but with fewer yellow segments, only three, and more red segments, which equal 5.

As demonstrated in Table 7, the suggested spectrophotometric approach produces a small number of red batches due to the single solvent used for analysis and little waste production. This method suggests that, in comparison to the reported methods, the suggested spectrophotometric approach is more environmentally friendly.

**Table 7:** Green evaluation of the suggested method using eco-scale and GAPI methods.

Parameters	Proposed spectrophotometric method	Reported method for RDV^22^	Reported method for MFX^31^
Reagents			
Ethanol	–	4	–
Methanol	6	–	–
Acetonitrile	–	4	–
0.1 N hydrochloric acid	–	–	4
Phosphate buffer	–	–	0
Acetone	–	–	6
Instruments			
Spectrophotometer			
Energy	0 [≤ 0.1 kWh/sample]	0 [≤ 0.1 kWh/sample]	0 [≤ 0.1 kWh/sample]
Occupational hazard	0	0	0
Waste	3	3	3
Total penalty points	Σ 9	Σ 11	Σ 13
Analytical eco-scale total score	91	89	87
Analytical eco-scale total score^a, b^	Excellent green analysis	Excellent green analysis	Excellent green analysis
GAPI pentagram			

^a^Analytical eco-scale total score = 100- total penalty points.

^b^If the score is better than 75, it means the green analysis is excellent. If the score exceeds 50, it means that the green analysis is acceptable. A score of 50 or less implies insufficient green analysis.

## Conclusion

In this work, we developed three green UV spectrophotometric methods to determine RDV and MFX in different matrices: AS, EXRS and AM approaches. All things considered, the three approaches were shown to be straightforward, precise, and devoid of complicated tools or procedures. They are appropriate for routine analysis of RDV and MFX in plasma since they are also selective and sensitive. The developed method’s compliance with green analytical chemistry principles was assessed by the utilization of the analytical eco-scale, and GAPI. Comparison of the developed methods with published spectrophotometric methods revealed that they adhered closely to the principles of green analytical chemistry.

## Data Availability

The data that support the findings of this study are available from the corresponding author, upon reasonable request.
